# Protective Effect of *Sargassum fusiforme* Fucoidan against Ethanol-Induced Oxidative Damage in In Vitro and In Vivo Models

**DOI:** 10.3390/polym15081912

**Published:** 2023-04-17

**Authors:** Lei Wang, Jae-Young Oh, Hye-Won Yang, Jimin Hyun, Ginnae Ahn, Xiaoting Fu, Jiachao Xu, Xin Gao, Seon-Heui Cha, You-Jin Jeon

**Affiliations:** 1 College of Food Science and Engineering, Ocean University of China, Qingdao 266003, China; 2 Food Safety and Processing Research Division, National Institute of Fisheries Science, Busan 46083, Republic of Korea; 3 Department of Marine Life Sciences, Jeju National University, Jeju 63243, Republic of Korea; 4 Department of Marine Bio Food Science, Chonnam National University, Yeosu 59626, Republic of Korea; 5 Department of Marine Bio and Medical Sciences, Hanseo University, Seosan-si 31962, Republic of Korea; 6 Marine Science Institute, Jeju National University, Jeju 63333, Republic of Korea

**Keywords:** *Sargassum fusiforme*, ethanol, oxidative stress

## Abstract

Our previous studies have evaluated the bioactivities of a fucoidan isolated from *Sargassum fusiforme* (SF-F). To further investigate the health benefit of SF-F, in the present study, the protective effect of SF-F against ethanol (EtOH)-induced oxidative damage has been evaluated in in vitro and in vivo models. SF-F effectively improved the viability of EtOH-treated Chang liver cells by suppressing apoptosis. In addition, the in vivo test results indicate that SF-F significantly and dose-dependently increased the survival rate of zebrafish treated with EtOH. Further research results show that this action works through decreasing cell death via reduced lipid peroxidation by scavenging intracellular reactive oxygen species in EtOH-stimulated zebrafish. These results indicate that SF-F effectively protected Chang liver cells and zebrafish against EtOH-induced oxidative damage and suggest the potential of SF-F to be used as an ingredient in the functional food industry.

## 1. Introduction

Ethanol (EtOH) is a water-soluble compound that easily penetrates the cell membrane. EtOH is the main component of alcoholic beverages. Excessive EtOH consumption causes cellular damage, which further leads to adverse effects on the liver, kidney, heart, intestine, brain, and stomach [1,2,3,4,5]. Based on the report from the World Health Organization in 2018, the death rates caused by alcohol consumption correspond to 5.1% of all deaths worldwide. In particular, excessive EtOH intake can cause many hepatocytes damage and diseases such as fatty liver, chronic hepatitis, alcoholic hepatitis, hepatic fibrosis, hepatic cirrhosis, as well as liver cancer [6,7,8,9]. Thus, protecting the body against EtOH-induced damage has taken the researcher’s attention.

Multiple reports suggested that EtOH-stimulated liver damage could be caused by oxidative stress [10,11,12,13,14]. Thus, a compound that possesses the effect of protecting the body against oxidative stress can be thought of as a potential ingredient for developing a therapeutic agent treatment for liver damage caused by EtOH intake. Seaweeds contain a lot of bioactive compounds such as phenolic compounds, carotenoids, fatty acids, dietary fibers, proteins, polysaccharides, and sterols [15,16,17,18,19,20,21,22,23]. These seaweed-derived compounds possess potent antioxidant activity [24,25]. In particular, fucoidans isolated from brown seaweeds were reported on their potent antioxidant effects [26,27,28,29]. Philipp et al. have extracted the fucoidans from five brown seaweeds and investigated their antioxidant effects [28]. The results suggest that these fucoidans effectively suppressed oxidative stress stimulated by hydrogen peroxide or tert-butyl hydroperoxide in retinal pigment epithelium [28]. Jayawardena et al. have evaluated the antioxidant effect of the *Padina boryana* fucoidan (PBP) and the results indicate that PBP remarkably suppressed Vero cells and zebrafish damage caused by hydrogen peroxide [30]. Lee et al. investigated the antioxidant activities of low-molecular-weight fucoidans isolated from the brown seaweed *Sargassum autumnale* [31]. The results indicate that the fucoidan isolated from *S. autumnale* (SAPF3) effectively scavenged free radicals and protected Vero cells and zebrafish against oxidative damage stimulated by hydrogen peroxide [31].

*Sargassum fusiforme*, an edible brown seaweed, has been consumed as herb medicine and food since ancient times. The fucoidan isolated from *S. fusiforme* has been reported on its several bioactivities [32,33]. Li et al. have reported the inhibitory effect of the fucoidan isolated from *S. fusiforme* on the occurrence and development of inflammation-related colorectal cancer [32]. The results indicate that the administration of the fucoidan isolated from *S. fusiforme* in mice can effectively prevent the development of colitis-associated colorectal cancer by inhibiting the expression of the inflammatory factors in the colonic tissues [32]. Zuo et al. have investigated the anti-obesity effect of the fucoidan isolated from *S. fusiforme* [33]. The results show that the fucoidan isolated from *S. fusiforme* (Fuc) remarkably suppressed obesity in high-fat-high-fructose-fed mice. Further data suggest that Fuc might remodel the gut microbiota and exert its weight loss and hypolipidemic effects via increasing energy expenditure [33].

In our previous studies, a fucoidan (SF-F) was isolated from the enzyme-assessed extract of *S. fusiforme* and the bioactivity of SF-F was evaluated. The results demonstrate that SF-F possesses strong anti-inflammatory, photoprotective, antioxidant, skin-whitening, and anti-wrinkle effects [34]. To further explore the bioactivity of SF-F, in the current research, the in vitro and in vivo protective effects of SF-F against EtOH-stimulated oxidative damage in Chang liver cells and in zebrafish were investigated.

## 2. Materials and Methods

### 2.1. Chemicals and Reagents

The seaweed was collected in June 2019 from the coastal area of Jeju Island, South Korea. Penicillin-streptomycin, Dulbecco’s modified Eagle medium, trypsin-EDTA, and fetal bovine serum were purchased from Gibco-BRL. 3-(4,5-Dimethylthiazol-2-yl)-2,5-diphenyltetrazolium bromide (MTT), 1,3-bis (diphenylphosphino) propane (DPPP), acridine orange (AO), dimethyl sulfoxide, 2,7-dichlorofluorescein diacetate (DCFH2-DA), and EtOH were purchased from Sigma-Aldrich Co (St. Louis, MO, USA).

### 2.2. Preparation and Characterization of SF-F

SF-F was prepared based on the protocol described in our previous study [35]. In brief, the seaweed *S. fusiforme* was hydrolyzed by the Celluclast (Sigma, St. Louis, MO, USA, ≥700 units/g). The extract was precipitated by ethanol and the crude *S. fusiforme* polysaccharides were obtained. The crude *S. fusiforme* polysaccharides were further purified by the DEAE-cellulose column and eluted by NaCl-containing sodium acetate. The purified polysaccharide (SF-F) was obtained. The carbohydrate and sulfate content of SF-F was determined. The neutral sugar component of SF-F was determined using high-performance anion-exchange chromatography with pulsed amperometric detection. The molecular weight of SF-F was analyzed using high-performance gel permeation chromatography.

### 2.3. Cell Culture

The Chang liver cells (ATCC^®^ CCL-13^TM^) were maintained in Dulbecco’s modified Eagle medium containing 10% fetal bovine serum and 1% penicillin-streptomycin. The Chang liver cells were sub-cultured every 3 days and seeded at a density of 1 × 10^5^ cells/mL for experiments. The Chang liver cells were seeded and incubated for 24 h for further treatment and analysis.

### 2.4. Measurement of Cytotoxicity and Cytoprotective Effect of SF-F

The cytotoxicity of SF-F on Chang liver cells was evaluated through an MTT assay. To evaluate the cytotoxicity of SF-F on Chang liver cells, the cells were seeded in a 24-well plate at a density of 1 × 10^5^ cells/mL for 24 h. After incubation, the Chang liver cells were treated with 12.5, 25, 50, 100, and 200 μg/mL SF-F, respectively. The control group was treated with the same volume of 1× PBS buffer. The Chang liver cells were incubated with different concentrations of SF-F for 24 h. After incubation, the SF-F-treated Chang liver cells were treated with 2 mg/mL MTT solution. After a 3 h reaction, the cell culture media was removed and the DMSO was added to each well. After formazan dissolved completely, the absorbance of the solution was detected using a microplate reader at a wavelength of 540 nm.

To investigate the cytoprotective effect of SF-F on Chang liver cells damaged by EtOH, the Chang liver cells were seeded in a 24-well plate at a density of 1 × 10^5^ cells/mL for 24 h. After incubation, the Chang liver cells were treated with 12.5, 25, and 50 μg/mL SF-F, respectively. After 1 h of incubation, the SF-F-treated Chang liver cells were stimulated with 5% EtOH and the cells were further incubated for 24 h. After incubation, the EtOH-stimulated Chang liver cells were treated with 2 mg/mL MTT solution. After 3 h, the cell culture media was removed and the DMSO was added to each well. After formazan dissolved completely, the absorbance of the solution was detected using a microplate reader at a wavelength of 540 nm.

### 2.5. Nuclear Staining with Hoechst 33342

To investigate the anti-apoptosis effect of SF-F, the Chang liver cells were seeded in a 24-well plate at a density of 1 × 10^5^ cells/mL for 24 h. After incubation, the Chang liver cells were treated with 12.5, 25, and 50 μg/mL SF-F for 1 h. Then, the SF-F-treated Chang liver cells were stimulated with EtOH. After 6 h of incubation, the EtOH-stimulated Chang liver cells were stained with Hoechst 33342 (1 mg/mL, stock) for 30 min. After the reaction, the apoptotic body was detected based on the protocol described by Wang et al. [35].

### 2.6. Application of EtOH to Zebrafish Embryos

The adult zebrafish were maintained according to the condition described previously [36]. Approximately 7~9 h post-fertilization, the zebrafish embryos were transferred to a 12-well plate and treated with 12.5, 25, and 50 μg/mL SF-F, respectively. The control group was treated with the same volume of 1x PBS buffer. The embryos were incubated with SF-F for 1 h. After incubation, the SF-F-treated zebrafish embryos were stimulated with EtOH (2%), and the zebrafish embryos were incubated with EtOH until 24 hpf. Then, the embryos were incubated with the fresh embryo media and the survival rates of EtOH-stimulated zebrafish were measured at 3 days post-fertilization by counting the number of live zebrafish. The surviving zebrafish were used for further experiments to investigate the level of ROS, cell death, and lipid peroxidation of EtOH-treated zebrafish.

### 2.7. Measurement of ROS Generation, Cell Death, and Lipid Peroxidation in Zebrafish

To investigate the ROS generation of EtOH-stimulated zebrafish, at 3 days post-fertilization, the zebrafish larvae were stained with DCFH2-DA for 1 h. After incubation, the zebrafish larvae were washed with fresh embryo media, and the individual zebrafish were photographed after anesthetization under a fluorescence microscope. To investigate the cell death of EtOH-stimulated zebrafish, at 3 days post-fertilization, the zebrafish larvae were stained with AO for 30 min, and the zebrafish larvae were washed with fresh embryo media. The individual zebrafish were photographed after anesthetization under a fluorescence microscope. To investigate the lipid peroxidation in EtOH-stimulated zebrafish, at 3 days post-fertilization, the zebrafish larvae were stained with DPPP for 3 h. After incubation, the zebrafish larvae were washed with fresh embryo media, and the individual zebrafish were photographed after anesthetization under a fluorescence microscope. The fluorescence intensity of individual zebrafish larvae was quantified using the ImageJ program.

### 2.8. Statistical Analysis

The experiments were performed in triplicates. The data were expressed as means ± standard errors, and one-way ANOVA was used to compare the mean values of each treatment in SPSS 20.0. Significant differences between the means were identified by Tukey’s test.

## 3. Results and Discussion

### 3.1. SF-F Protects Chang Liver Cells against Oxidative Stress Stimulated by EtOH

EtOH has direct hepatotoxicity. It can cause steatosis at the beginning and develop into alcoholic liver diseases in the later stage, such as alcoholic hepatitis, hepatic fibrosis, hepatic cirrhosis, as well as liver cancer [1,11]. Oxidative stress is an important event in the development of EtOH-induced liver damage [10]. Therefore, regulation of oxidative stress has been thought of as a strategy to protect the liver against EtOH-induced damage and diseases in medicine and functional food research areas.

Natural products possess various advantages such as low side effects and remarkable effects. Currently, finding a natural product and developing it as an ingredient in the medicine, cosmetic, and functional food industries has taken researchers’ attention. Chen et al. have investigated the protective effect of docosahexaenoic acid against liver cell damage stimulated by hydrogen peroxide [37]. The results indicate that docosahexaenoic acid could scavenge intracellular ROS stimulated by hydrogen peroxide and stimulate the cellular antioxidation response in AML12 cells [37]. Eva Ari Wahyuni et al. have evaluated the effect of propolis on oxidative DNA damage stimulated by 4-aminobiphenyl in human liver cells [38]. The results demonstrate that propolis effectively decreased oxidative DNA damage by reducing intracellular ROS levels in human liver cells [38].

Fucoidans isolated from seaweeds possess potent antioxidant activity and have a hepatoprotective potential. The antioxidant activities of *S. fusiforme* fucoidans have been reported in previous studies [35,39,40]. In our previous studies, a fucoidan (SF-F) was isolated from the enzyme-assessed extract of *S. fusiforme,* and its antioxidant activity was evaluated [35]. The results indicate that SF-F contains 99.01% fucoidan that constitutes 71.79% carbohydrate and 27.22% sulfate contents. The neutral sugar component analysis results indicate that SF-F is composed of 79.20% fucose, 2.09% rhamnose, 0.19% glucose, 0.38% arabinose, and 18.13% mannose. The molecular weight analysis result shows that SF-F has a molecular weight of 102.67 kDa. Furthermore, the bioactivity analysis results indicate that SF-F effectively attenuated oxidative stress stimulated by hydrogen peroxide in vitro in Vero cells and in vivo in zebrafish [35]. These results demonstrate the antioxidant activity of SF-F and suggest its hepatoprotective potential. Therefore, in the present study, the effect of SF-F on EtOH-stimulated oxidative damage was investigated in in vitro and in vivo models.

**Figure 1:**
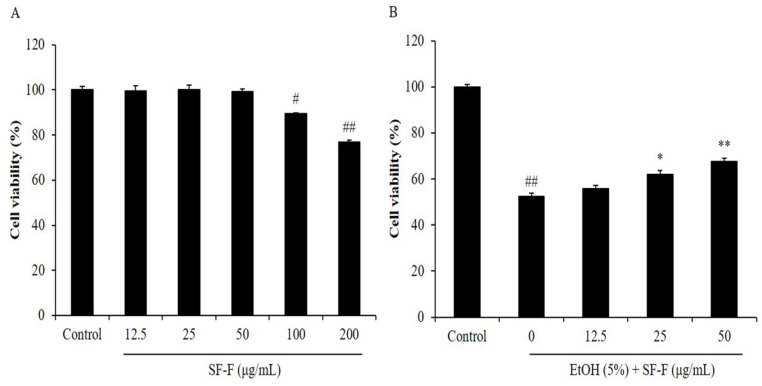
Protective effect of SF-F against EtOH-stimulated Chang liver cells death. (**A**) Cytotoxicity of SF-F on Chang liver cells; (**B**) cytoprotective effect of SF-F in EtOH-stimulated Chang liver cells. Cell viability was measured by MTT assay. The data are expressed as the mean ± SE. * *p* < 0.05 and ** *p* < 0.01 as compared to the EtOH-treated group; ^#^ *p* < 0.05 and ^##^ *p* < 0.01 as compared to the control group.

EtOH-treated Chang liver cells were used to evaluate the hepatoprotective and antioxidant effects of seaweed-derived compounds [36]. Thus, in the present study, we selected EtOH-stimulated Chang liver cells to evaluate the in vitro protective effect of SF-F on EtOH-stimulated oxidative stress. In the current study, the cytoprotective effect of SF-F in EtOH-stimulated Chang liver cells was investigated. As Figure 1A shows, the viabilities of Chang liver cells treated with 100 and 200 μg/mL SF-F were 89.53 and 76.82% compared to the control group (100%), respectively. However, the viabilities of Chang liver cells treated with 12.5, 25, and 50 μg/mL SF-F were all close to 100% (Figure 1A). These results show that SF-F possesses slight cytotoxicity to Chang liver cells at a concentration higher than 100 μg/mL. However, it is non-toxic to Chang liver cells at a concentration below 50 μg/mL. Thus, the highest concentration of SF-F applied to Chang liver cells was determined as 50 μg/mL in further studies.

**Figure 2:**
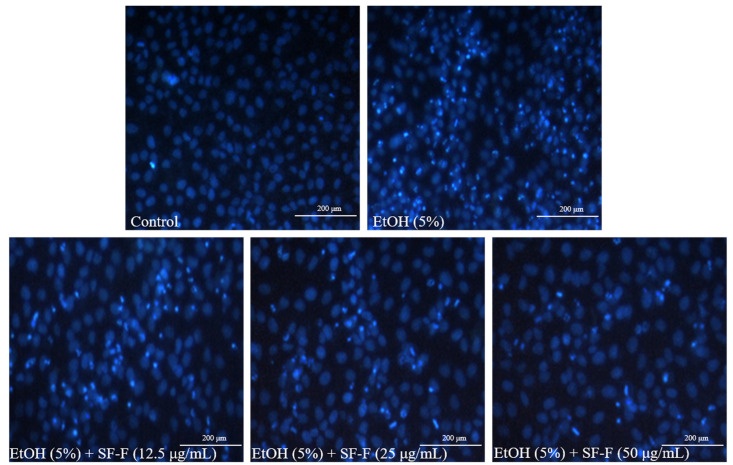
Protective effect of SF-F against EtOH-induced apoptosis in Chang liver cells. The apoptotic body formation was observed under a fluorescence microscope after Hoechst 33342 staining.

As Figure 1B shows, the viability of EtOH-treated Chang liver cells was significantly decreased to 52.48% compared to non-treated cells (100%), whereas the viabilities of EtOH-stimulated Chang liver cells were increased to 55.88, 62.05, and 67.78% after being treated with 12.5, 25, and 50 μg/mL SF-F, respectively (Figure 1B). These results indicate that EtOH significantly induced cell death of Chang liver cells, and SF-F effectively protected Chang liver cells against EtOH-stimulated cell death in a concentration-dependent manner. In addition, EtOH significantly induced apoptosis in Chang liver cells. However, the apoptosis body amount was remarkably reduced by SF-F treatment (Figure 2). These results show that SF-F effectively suppressed apoptosis stimulated by EtOH in Chang liver cells, and the effect was shown in a concentration-dependent manner.

Kang et al. investigated the protective effect of the ethanol extract from the brown seaweed *Ecklonia cave* against EtOH-stimulated Chang liver cells [36]. The results indicate that EtOH effectively decreased the viability of Chang liver cells by stimulating apoptosis. The ethanol extract containing polyphenol remarkably protected Chang liver cells against EtOH-induced oxidative damage by improving the viability of EtOH-treated Chang liver cells via suppressing apoptosis in a concentration-dependent manner [36]. Compared to the previous and present study, we can speculate that SF-F significantly and concentration-dependently protected Chang liver cells against EtOH-stimulated cell death by suppressing apoptosis.

### 3.2. SF-F Protects Zebrafish against Oxidative Damage Stimulated by EtOH

Zebrafish are an extensively studied vertebrate model organism. It has several advantages, such as the similarity of their genome to mammals, their short life span and comparatively small size, and the ability of the females to produce a large number of eggs. Owing to these advantages, zebrafish are thought of as an ideal in vivo model used in biological, pharmaceutical, and cosmeceutical studies. EtOH-stimulated zebrafish were used to investigate the in vivo damage induced by EtOH in previous studies [36,41,42]. Kang et al. reported that EtOH significantly stimulated oxidative damage in zebrafish displayed in decreasing the survival rate, stimulating ROS production, and inducing cell death [36]. These adverse effects can effectively be suppressed by the seaweed-derived phenolic compound [36]. Thus, zebrafish exposed to EtOH were selected to investigate the protective effect of SF-F against EtOH-stimulated in vivo oxidative damage in this study.

**Figure 3:**
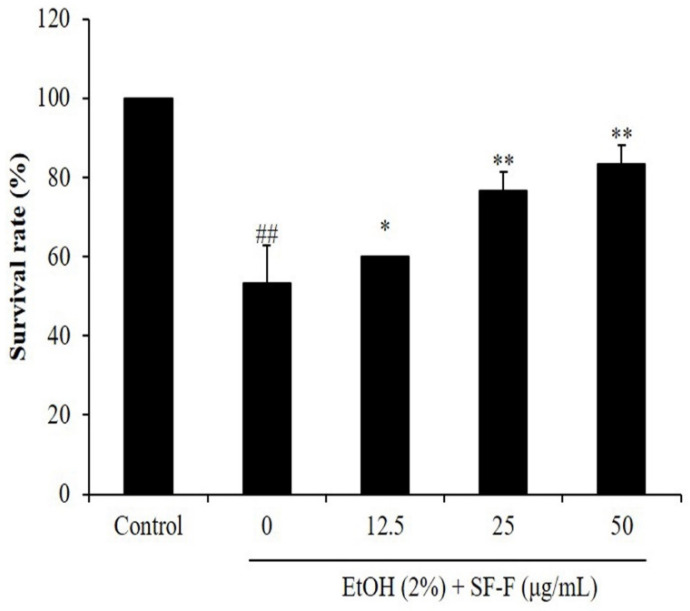
The survival rate after pretreatment with SF-F and/or co-treatment with EtOH in zebrafish. The data are expressed as the mean ± SE. * *p* < 0.05, ** *p* < 0.01 as compared to the EtOH-treated group and ^##^ *p* < 0.01 as compared to the control group.

**Figure 4:**
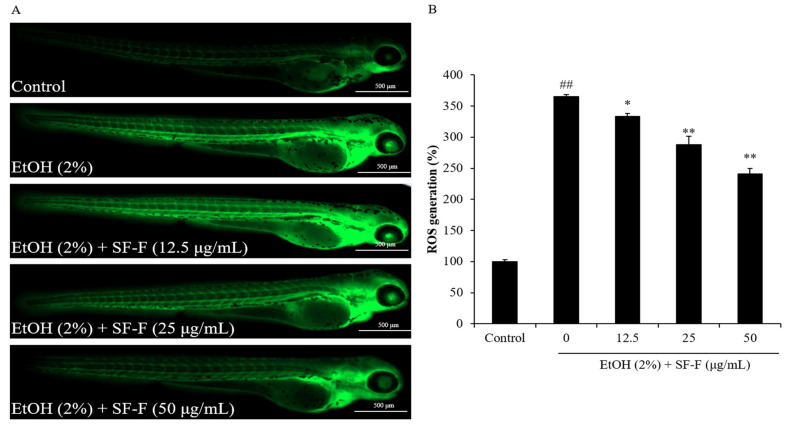
The protective effect of SF-F during EtOH-induced ROS production in zebrafish. (**A**) Zebrafish under fluorescence microscope; (**B**) the levels of ROS generation. ROS was detected by DCFH2-DA staining and ROS levels of EtOH-treated zebrafish were measured by ImageJ software. The experiments were conducted in triplicate, and the data are expressed as the mean ± SE (*n* = 3). * *p* < 0.05, ** *p* < 0.01 as compared to the EtOH-treated group and ^##^ *p* < 0.01 as compared to the control group.

In the current research, the in vivo protective effect of SF-F against EtOH-induced oxidative damage was investigated through an evaluation of the ROS level, survival rate, cell death, and lipid peroxidation in the EtOH-stimulated zebrafish. As Figure 3 shows, the survival rate of EtOH-treated zebrafish decreased to 53.33% from 100% compared to the control group. However, SF-F effectively increased the survival rate of zebrafish to 60, 76.67, and 83.33% at doses of 12.5, 25, and 50 μg/mL, respectively (Figure 3). These results indicated that EtOH significantly reduced the survival rate of zebrafish, and SF-F could improve the survival rate of EtOH-treated zebrafish in a dose-dependent manner. As the results show, the intracellular ROS level of EtOH-stimulated zebrafish increased to 365.68% from 100% (the control group). However, the intracellular ROS level of EtOH-treated zebrafish was reduced to 333.42, 287.76, and 240.77% by SF-F at a dose of 12.5, 25, and 50 μg/mL, respectively (Figure 4). These results indicate that EtOH significantly stimulated ROS generation in zebrafish, and the ROS levels of EtOH-treated zebrafish were remarkably reduced by SF-F treatment in a dose-dependent manner.

**Figure 5:**
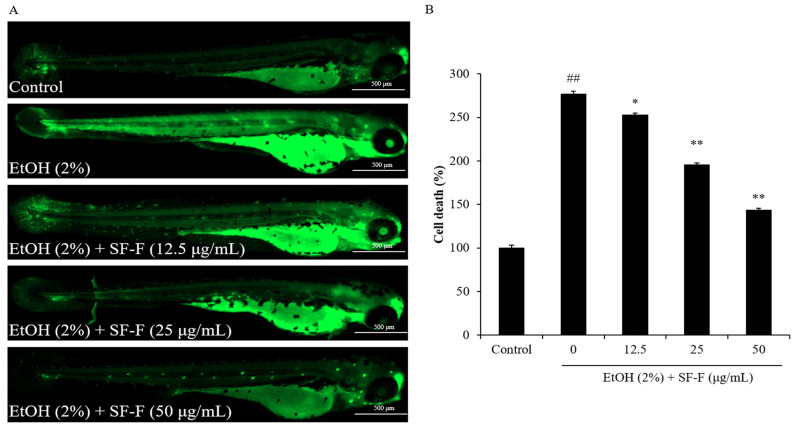
The protective effect of SF-F during EtOH-induced cell death in zebrafish. (**A**) Zebrafish under fluorescence microscope; (**B**) the measured levels of cell death. Cell death was detected by AO staining and cell death levels of EtOH-treated zebrafish were measured by ImageJ software. The experiments were conducted in triplicate, and the data are expressed as the mean ± SE (*n* = 3). * *p* < 0.05, ** *p* < 0.01 as compared to the EtOH-treated group and ^##^ *p* < 0.01 as compared to the control group.

In addition, EtOH induced a 176.79% increase in cell death in zebrafish compared to the control group, and SF-F reduced 24.18, 81.63, and 132.64% cell death in EtOH-stimulated zebrafish at a dose of 12.5, 25, and 50 μg/mL, respectively (Figure 5). These results show that EtOH significantly stimulated cell death in zebrafish, and SF-F effectively suppressed cell death stimulated by EtOH in a dose-dependent manner.

**Figure 6:**
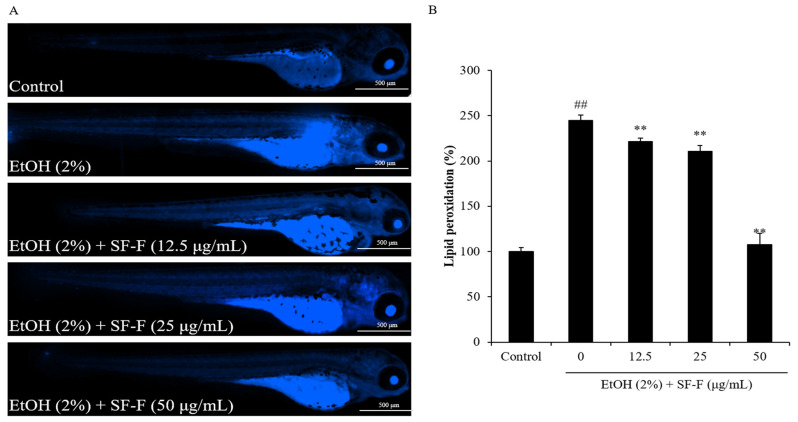
The protective effect of SF-F during EtOH-induced lipid peroxidation in zebrafish. (**A**) Zebrafish under fluorescence microscope; (**B**) the measured levels of lipid peroxidation. Lipid peroxidation was detected by DPPP staining and lipid peroxidation levels of zebrafish were measured by ImageJ software. The experiments were conducted in triplicate, and the data are expressed as the mean ± SE. ** *p* < 0.01 as compared to the EtOH-treated group and ^##^ *p* < 0.01 as compared to the control group.

Furthermore, as Figure 6 shows, the lipid peroxidation level of EtOH-treated zebrafish was increased to 244.74% compared to the control group (100%). However, the lipid peroxidation levels of EtOH-treated zebrafish treated with 12.5, 25, and 50 μg/mL SF-F were decreased to 221.45, 210.42, and 107.50%, respectively (Figure 6). These results demonstrate that SF-F remarkably inhibited EtOH-stimulated lipid peroxidation in a dose-dependent manner.

The previous results suggest that hydrogen peroxide could stimulate cell death by inducing lipid peroxidation via stimulating intracellular ROS production in zebrafish. These adverse effects could suppressed by algal polysaccharides [31]. Compared to the previous and current results, we can speculate that SF-F effectively protected zebrafish against EtOH-induced oxidative damage by suppressing cell death via reducing lipid peroxidation by scavenging intracellular ROS.

## 4. Conclusions

In the current research, the protective effect of SF-F against EtOH-stimulated oxidative damage was evaluated in in vitro and in vivo models. The results demonstrate that SF-F remarkably protected Chang liver cells against oxidative stress stimulated by EtOH via inhibiting apoptosis by scavenging intracellular ROS. Furthermore, the in vivo test results indicate that SF-F significantly suppressed EtOH-stimulated oxidative damage by suppressing cell death via reducing lipid peroxidation by scavenging intracellular ROS in zebrafish. These results show that SF-F remarkably protected Chang liver cells and zebrafish against EtOH-stimulated oxidative damage and suggest the potential of SF-F to be used as an ingredient in the functional food industry.

## Data Availability

Data is contained within the article.
